# Calcitonin Gene-Related Peptide Level in Cystic Fibrosis Patients

**DOI:** 10.3390/life14050565

**Published:** 2024-04-27

**Authors:** Sabina Galiniak, Marek Biesiadecki, Iwona Rościszewska-Żukowska, Marta Rachel

**Affiliations:** 1 Institute of Medical Sciences, Medical College, Rzeszów University, Warzywna 1a, 35-310 Rzeszów, Poland; mbiesiadecki@ur.edu.pl (M.B.); iwona.rosciszewska@op.pl (I.R.-Ż.); rachel@popia.pl (M.R.); 2 Department of Allergology and Cystic Fibrosis, State Hospital 2 in Rzeszow, Lwowska 60, 35-301 Rzeszów, Poland

**Keywords:** calcitonin gene-related peptide, cystic fibrosis, CFTR mutation

## Abstract

Calcitonin gene-related peptide (CGRP) has long been implicated in both the physiology and pathophysiology of the respiratory tract. The objective of our study was to determine the serum concentration of alpha CGRP (αCGRP) in cystic fibrosis (CF) that arises from mutations in the gene responsible for encoding the cystic fibrosis transmembrane conductance regulator (CFTR) protein. Currently, there are not many data in the literature about the role of CGRP in CF. The serum level of αCGRP was estimated using the enzyme-linked immunosorbent assay among 64 patients with CF and 31 healthy controls. The αCGRP concentration in the CF group was 62.51 ± 15.45 pg/mL, while in the control group it was 47.43 ± 8.06 pg/mL (*p* < 0.001). We also compared the level of αCGRP in CF patients according to the type of CFTR mutation. Homozygotes for ΔF508 had higher αCGRP levels than heterozygotes (67.9 ± 10.2 vs. 54.5 ± 18.3 pg/mL, *p* < 0.01). The level of this neuropeptide was statistically higher in patients with severe disease than in those with mild CF (*p* = 0.003) when patients were divided into three groups by spirometry results. αCGRP concentration was not correlated with age, sex, clinical parameters, and pulmonary function test results in the study participants. The results of our study suggest a significant increase in the concentration of αCGRP in the serum of patients with CF compared to the control group. This observation opens interesting possibilities for understanding the role of αCGRP in the context of CF pathophysiology.

## 1. Introduction

Calcitonin gene-related peptide (CGRP) is a 37-amino acid neuropeptide, featuring an N-terminal disulfide bond and a C-terminus that is amidated. It exhibits broad distribution within discrete regions of both the central and peripheral nervous systems, indicating potential involvement in cardiovascular, integrative, and gastrointestinal functions [1]. Furthermore, CGRP is a remarkably potent vasodilator and, to some extent, harbors protective mechanisms vital for both physiological and pathological conditions concerning the cardiovascular system and wound healing [2,3]. It is hypothesized that CGRP also plays a role in arthritis, skin conditions, diabetes, and obesity [4]. Moreover, CGRP is recognized as one of the fundamental factors in the pathophysiology of migraine [5]. Most neuropeptides, including CGRP, are also released from endocrine cells and can act on both neural and nonneural targets [2]. CGRP is present throughout the respiratory tract and CGRP receptors are expressed on many cells, including pulmonary artery cells, epithelial goblet cells, and innate lymphoid immune cells [6]. Cystic fibrosis (CF) is an autosomal recessive genetic disorder resulting from mutations in the CF transmembrane conductance regulator (CFTR) gene responsible for encoding a chloride channel. The deletion of phenylalanine at position 508 (ΔF508) represents the most prevalent mutation among CF patients. The functionality of this chloride channel plays a critical role in maintaining the osmotic balance of mucus and regulating its viscosity. The imbalance or lack of fluids and ions resulting from duct dysfunction causes the production of thick and viscous mucus, leading to dysfunction of the exocrine glands [7]. Neural involvement in CF has been also proposed [8].

It is noteworthy that elevated levels of CGRP have been detected in the submucosal glands of the airways in humans with CF, as well as in CF animal models including mice, ferrets, and pigs [9]. This suggests that the increased expression of CGRP in CF submucosal glands may represent a compensatory mechanism aimed at restoring CFTR-dependent secretions [8,9]. Within the CGRP family, the two most closely related peptides are α-CGRP and β-CGRP, which are expressed from two genes located at different sites on chromosome 11 in humans. α-CGRP may be the predominant form secreted by neurons or endocrine cells, is well studied, and has important physiological functions [1,2,3,4]. 

Despite the significant implications of αCGRP in various physiological and pathological processes, there remains a notable gap in our understanding regarding its serum levels, specifically in patients diagnosed with CF. This lack of data underscores the need for comprehensive investigation to elucidate the role of αCGRP in the context of CF pathology. Thus, the primary objective of this study was to bridge this knowledge gap by quantifying and evaluating the serum concentration of αCGRP in individuals diagnosed with CF. Furthermore, we sought to explore the potential correlations between αCGRP levels and various clinical parameters characteristic of CF. These parameters may encompass but are not limited to pulmonary function tests, nutritional status, microbial colonization patterns, and disease severity indices. By examining these associations, we aimed to shed light on the potential clinical relevance of αCGRP in the context of CF pathophysiology and disease progression.

## 2. Materials and Methods

### 2.1. Ethical Issues

The study protocol, as outlined in resolution no. 2022/023 by the Bioethics Committee of Rzeszów, underwent thorough scrutiny and received official approval. In accordance with the principles set forth in the Declaration of Helsinki, the research was meticulously conducted, guided by the relevant guidelines and regulations governing scientific inquiry. Prior to commencement, all prospective participants, or their legal guardians in the case of minors or individuals unable to provide consent, were fully informed about the nature and objectives of the study. 

### 2.2. Study Group

A cross-sectional study was conducted at a single center involving a sample of 64 CF patients and 31 healthy controls. Participants were recruited from the local CF clinic between February and October 2021. We recruited patients aged 9–39 with a confirmed diagnosis of CF based on current diagnostic criteria [10,11]. The next criteria for enrolling patients in the study were the following: forced expiratory volume in the first second (FEV_1_) greater than 35% of predicted stable pulmonary disease as defined by both clinical impressions and no hospitalizations in the 1 month before recruitment to the study. Exclusion criteria were heart and liver failure, migraine, psychiatric disorder, diabetes mellitus, having undergone solid organ transplantation, disease exacerbation, chronic immunosuppressive and corticosteroids or antibiotic treatment. Furthermore, patients were excluded if they were unable to perform spirometry or declined to participate in the study. Consequently, six CF patients were excluded from the study.

All CF patients suffered from pancreatic insufficiency and received pancreatic enzyme replacement therapy, human DNase I recombinant, fat-soluble vitamins in the form of ADEK tablets, and inhalation of 3–7% sodium chloride 3–4 times daily as recommended [12]. It is worth adding that patients with CF were not treated with CFTR modulators because, at the time of the study, this treatment was not yet financed in Poland. On the day of study, we recorded and estimated the following clinical variables in all participants: sex, age, body mass index (BMI), type of CFTR mutations, blood morphology and serum analysis, and sputum microbiology. Data on the type of mutation and chronic bacterial infection were obtained from current hospital records. CF subjects were additionally stratified into three groups according to the severity of their disease as determined by the results of FEV_1_: mild disease (FEV_1_ > 75% predicted), moderate disease (FEV_1_ ranging from 45% to 75% predicted), and severe disease (FEV_1_ < 45% predicted), as previously outlined in [13].

Healthy controls were recruited concurrently from the local clinic. The control cohort comprised volunteers matched for age and sex who were devoid of any medical history or findings indicating disease upon physical examination. These volunteers abstained from medication, including supplements, for a period of 30 days preceding the study. All individuals in the control group exhibited normal pulmonary function test results, along with standard findings in biochemical and hematological assays. Among the participants from the control group and patients with CF, there were no people with skin diseases, arthritis, other respiratory diseases such as asthma, central nervous system disorders, or wound healing disorders. The body mass index was calculated as a person’s weight in kilograms divided by the square of height in meters (kg/m^2^).

### 2.3. Spirometry

To ensure consistency and accuracy in assessing pulmonary function, spirometry was conducted for all subjects. Utilizing a standard spirometry device (Lungtest 1000, MES, Kraków, Poland), in accordance with established guidelines [14], pulmonary function was systematically evaluated. The mean value of FEV_1_ over the last six months was calculated for each participant and expressed as a percentage of the predicted value based on age and sex. This approach enables normalization of lung function data, facilitating meaningful comparisons across individuals and groups while accounting for variations attributable to age and sex differences.

### 2.4. Blood Sampling

Following an overnight fasting period, peripheral blood samples were collected, with 5 mL drawn from each participant in the morning to minimize potential confounding factors. These blood samples were promptly transferred into specialized blood collection tubes. Subsequently, the collected blood samples underwent centrifugation at 1500× *g* for 10 min at 4 °C. The obtained serum was then carefully aliquoted into individual storage vials. These aliquots were promptly stored at a temperature of −80 °C. Serum samples were not stored for more than one month to mitigate the effects of prolonged storage on sample quality. Furthermore, to prevent repeated freeze–thaw cycles that could compromise sample integrity, serum aliquots were thawed only once at room temperature immediately prior to analysis.

### 2.5. Blood Counts and Serum Analysis

Blood morphology was performed using a hematology analyzer (Siemens Healthineers, Erlangen, Germany). The concentration of C-reactive protein (CRP) was estimated using the dry chemistry immunological method on a VITROS 250 analyzer (Ortho Clinical Diagnostics, Johnson and Johnson, Rochester, NY, USA). Immunoturbidimetric assays were used for the determination of IgG.

### 2.6. αCGRP

αCGRP serum concentrations were measured in duplicates using the enzyme-linked immunosorbent assay (ABclonal Biotechnology Co., Ltd., Wuhan, China) according to the manufacturer’s protocol. The limit of detection for αCGRP was 5.35 pg/mL, and the within-assay and between-assay coefficients of variations were lower than 10% and lower than 12%, respectively. The absorbance was measured at the appropriate wavelength using a Tecan Infinite 200 PRO multimode reader (Tecan Group Ltd.; Männedorf, Switzerland). Serum levels of αCGRP were expressed in pg/mL.

### 2.7. Statistical Analysis

All statistical analyses were conducted using the STATISTICA software package (version 13.3, StatSoft Inc. 2017, Tulsa, OK, USA). Quantitative variables were reported as mean ± standard deviation (SD) and range. Qualitative variables were also reported as percentages. Comparisons of the groups were performed with the Mann–Whitney U test or Kruskal–Wallis test. Spearman’s rank correlation coefficient analysis was used to estimate the associations between the CGRP and clinical parameters, assuming linear dependence. *p*-values of less than 0.05 were considered to be statistically significant.

## 3. Results

A total of 42 females (65.7%) and 22 males with CF and 20 healthy females (64.5%) and 11 healthy males were included in the study. The characteristics of the study groups are presented in Table 1.

There was no difference in the age of the study groups, but CF patients had significantly lower BMI (*p* < 0.001). Patients were classified into two groups by genotypes: homozygous for ΔF508 (59.4%) and heterozygotes (40.7%). There was no difference in white blood cell and neutrophil counts, but CRP was significantly elevated in CF patients (*p* < 0.05). The FEV_1_ values in patients with CF were significantly worse than in healthy controls (*p* < 0.05). In total, 18 (28.1%) CF patients were positive for *Pseudomonas aeruginosa* and 20 (31.25%) patients were infected with *Staphylococcus aureus*, while 26 (40.65%) were uninfected. Analysis of the results of spirometry allowed for the division of patients with CF according to the severity of the disease: mild (n = 25, 39.1%), moderate (n = 19, 29.7%), and severe (n = 20, 31.2%).

**Figure 1:**
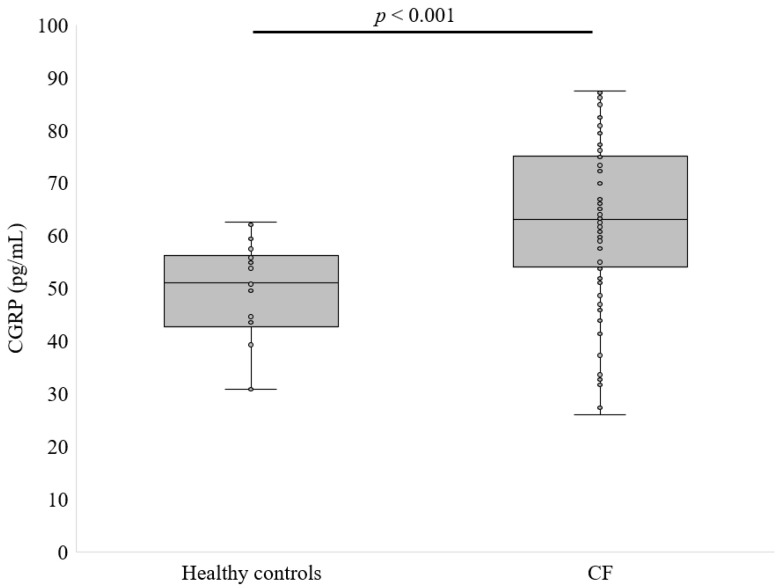
Level of CGRP in patients with CF compared to healthy participants (differences between means were analyzed using Mann–Whitney U tests).

Figure 1 presents the concentration of αCGRP among patients with CF and healthy subjects. 

**Figure 2:**
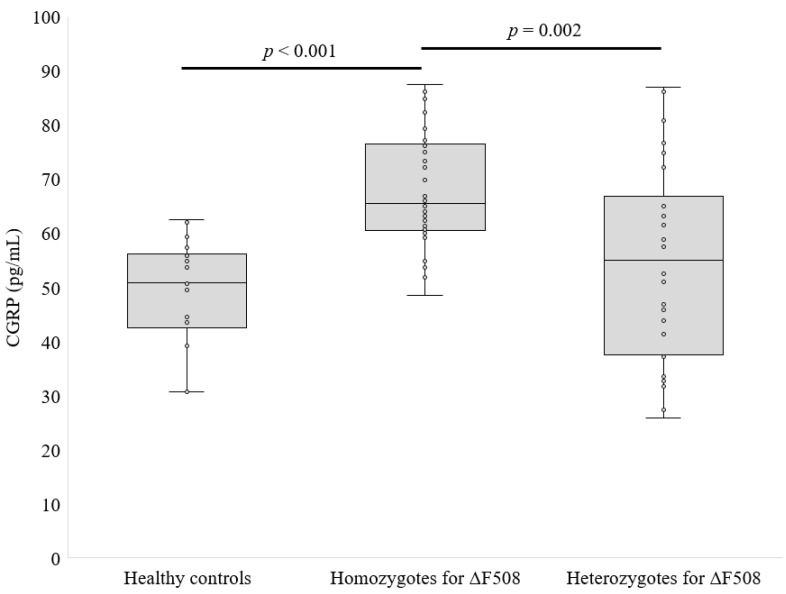
CGRP level by the type of *CFTR* mutations (differences between means were analyzed using Kruskal–Wallis U tests).

The αCGRP concentration in the CF group was 62.51 ± 15.45 pg/mL, while in the control group it was 47.43 ± 8.06 pg/mL (*p* < 0.001, Figure 1). The next step of this study was to check whether the type of CFTR mutation and the sex of the patients had an impact on the level of CGRP. Homozygotes for ΔF508 had higher αCGRP levels than heterozygotes (67.9 ± 10.2 vs. 54.5 ± 18.3 pg/mL, *p* < 0.01, Figure 2). However, there was no statistical difference in αCGRP concentrations between females and males with CF (63.5 ± 14.8 vs. 60.6 ± 10.7 ng/mL, *p* = 0.666, differences between means were analyzed using Mann–Whitney U tests).

The type of chronic bacterial infection did not affect αCGRP levels (CF patients with *P. aeruginosa*: 64.22 ± 12.6 pg/mL; CF patients with *S. aureus*: 69.1 ± 11.6 pg/mL; uninfected group: 56.24 ± 17.2 pg/mL; *p* = 0.063; differences between means were analyzed using Kruskal–Wallis U tests), although participants infected with *P. aeruginosa* or *S. aureus* had slightly higher concentrations of this neuropeptide. The αCGRP level in patients with mild disease was 55.44 ± 14.81 pg/mL, in patients with moderate disease it was 61.37 ± 15.82 pg/mL, and in patients with severe disease it was 65.31 ± 11 pg/mL. The level of this neuropeptide was statistically higher in patients with severe disease than in those with mild CF (*p* = 0.003; differences between means were analyzed using Kruskal–Wallis U tests).

Another important aspect of our study was to assess whether patients’ clinical parameters were correlated with αCGRP. Table 2 shows the correlation coefficients and the *p*-values estimated using Spearman correlation. αCGRP concentration was not correlated with BMI, CRP, IgG, and FEV_1_. At the same time, the results of the correlation between CGRP and age, white blood cell count, and neutrophil count, although not reaching statistical significance, are close to *p* = 0.05, indicating that further analysis is required across a larger number of CF patients.

## 4. Discussion

The results from our study present significant differences in the concentration of αCGRP present within the serum of patients diagnosed with CF when juxtaposed against levels in the control group, underscoring the potential significance of this neuropeptide in the context of CF pathophysiology.

CGRP has long been suggested to participate in physiology and pathophysiology of the respiratory tract. A large number of studies in recent years have provided direct evidence for an important modulatory role of CGRP in airway functions such as bronchial smooth muscle tone or vasoregulation [15]. The most prominent effects of CGRP in the airways are vasodilation and, in a few instances, bronchoconstriction. Moreover, a further pulmonary effect of CGRP is the induction of eosinophil migration and the stimulation of β-integrin-mediated T cell adhesion to fibronectin at the site of inflammation [16]. By contrast, CGRP inhibits macrophage secretion and the capacity of macrophages to activate T cells, indicating a potential anti-inflammatory effect [17]. Since CGRP participates in goblet cell hyperplasia and muc5AC induction in other disease models, it is possible that this neuropeptide is also involved in CF exacerbations, in which elevated muc5AC has been noted [18,19]. Furthermore, CC motif chemokine ligand 17 stimulates the release of greater amounts of CGRP than other inflammatory cytokines through a CCR4-dependent mechanism that has been implicated in other respiratory diseases [20]. As a result, CGRP can limit the activation of immune cells and reduce the production of inflammatory mediators. In cases of hypoxia, airway stem cells respond by proliferating and differentiating into neuroendocrine cells that secrete CGRP [21]. Episodes of hypoxia occur frequently in patients with CF, which may also explain the increased concentration of this neuropeptide in sick people [22]. Moreover, a study by Chang et al. proved that CGRP may be important in the mechanism of chronic cough [23]. Cough is part of the daily life of patients with CF and its most common symptom, which may also be related to the increased concentration of this neuropeptide [24].

CGRP can be present in a variety of body tissues, where it plays tissue-specific activities. The main source of serum CGRP is believed to be from the perivascular nerve endings [3] and it is elevated in certain pathological states, such as sepsis [25]. A recent study showed that lower levels of CGRP should negatively impact the respiratory physiology of COVID-19 patients due to vasoconstriction, improper angiogenesis, less epithelial repair, and faulty immune response [17]. On the other hand, patients with pulmonary hypertension had higher plasma CGRP than patients with normal pressure [26]. There are not many data in the literature about the role of CGRP in CF. An interesting aspect is the αCGRP concentration in CF patients, which was 62.51 ± 15.45 pg/mL in our study and 346.54 ± 47.19 pg/mL in a recent study by Al-Keilani et al. [27]. This may be due to the specificity of the test that was used to determine αCGRP or differences in the studied groups. Furthermore, Al-Keilani et al. studied CF exacerbation, and this may be another reflection of how CGRP levels reflect disease severity.

Previous studies have shown increased levels of CGRP in the submucosal glands of the respiratory tract in humans with CF, as well as in mice, ferrets, and pigs with CF, indicating a potential role for CGRP as a compensatory mechanism to restore CFTR-dependent secretion [7,28]. As we observed higher αCGRP concentrations in participants with CF, our results also seem to support these observations.

One interesting aspect of our results is the difference in αCGRP levels between patients homozygous and heterozygous for the ΔF508 mutation. Homozygous individuals for the ΔF508 mutation had significantly higher αCGRP levels. This suggests that different types of CFTR mutations may affect αCGRP production. However, more detailed research is needed to decipher this relationship.

In our study, αCGRP levels were higher in patients infected with *P. aeruginosa* or *S. aureus* than in uninfected participants. Similarly, serum CGRP level was significantly associated with positive *S. aureus* microbiology tests (*p* = 0.046) and the type of antibiotic therapy (*p* = 0.012) in a recent study by Al-Keilani et al., who stated that serum levels of αCGRP may predict response to antibiotic therapy in CF patients with pulmonary exacerbations [27]. Moreover, El Karim et al. proved that CGRP displayed antimicrobial activity against several types of bacteria, including *P. aeruginosa* [29].

In our study, there were no correlations between αCGRP levels and sex, age, or other clinical parameters among CF patients. In healthy subjects, it has been shown that plasma CGRP levels in women are significantly higher than in men [30,31], suggesting a direct relationship between estrogen levels and CGRP concentrations in blood. Due to the fact that our population included both children and adults, we did not observe this association. 

The results of the correlation of CGRP with age, as well as with counts of white blood cells and neutrophils, are close to the assumed level of statistical significance, so the cohort is also too small to reject a correlation between CGRP and these parameters in CF. These results, although not statistically significant, may indicate the need for further studies in larger numbers of CF patients to clearly determine the associations between CGRP and age and hematological results. Specifically, a correlation between CGRP and age may reflect the association of CGRP with CF disease severity for CF disease progression. White blood cell count or neutrophil counts may correlate with CGRP in CF, but not in healthy controls. No correlation between CGRP and white blood cell and neutrophil counts was observed in hospitalized acute COVID-19 patients [32]. In addition to being synthesized by nerves, CGRP is also produced by immune cells, including lymphocytes, monocytes, and macrophages [16]. Furthermore, within the bone marrow, CGRP can act on hematopoietic progenitors [33].

CGRP can indeed be considered an inflammation marker, at least in certain aspects. While traditionally recognized for its role in vasodilation and nociception, emerging research suggests that CGRP is intricately involved in modulating inflammatory responses. Studies have shown that CGRP levels increase in response to various inflammatory stimuli. For example, in conditions such as rheumatoid arthritis, inflammatory bowel disease, and asthma, elevated levels of CGRP have been observed in affected tissues or systemic circulation [34,35]. This suggests that CGRP production is upregulated as part of the inflammatory response. Moreover, by dilating blood vessels, CGRP promotes increased blood flow to inflamed tissues, facilitating the delivery of immune cells and mediators to the site of inflammation. CGRP influences the function of immune cells involved in inflammation, including T cells, macrophages, and dendritic cells [15,36,37].

While our study provides valuable insights into the association between αCGRP and CF, it is imperative to acknowledge the inherent limitations that may impact the generalizability and depth of our findings. One such limitation pertains to the relatively modest sample size employed in this study. Although our study cohort was carefully selected and rigorously evaluated, the inclusion of a larger and more diverse patient population would strengthen the statistical power and robustness of our conclusions. By encompassing a broader spectrum of individuals with CF, including those with varying genetic backgrounds, future studies can offer a more comprehensive understanding of the role of αCGRP in CF pathogenesis. Additionally, the absence of long-term follow-up data represents another noteworthy limitation of our study. Longitudinal assessment over an extended duration would offer valuable insights into the dynamic changes and prognostic implications of αCGRP expression in CF progression.

## 5. Conclusions

A compelling association emerged between the severity of CF and αCGRP levels, with patients exhibiting severe manifestations of the disease demonstrating markedly elevated levels of this neuropeptide compared to those with milder forms of CF. This correlation underscores the potential role of αCGRP as a biomarker reflective of disease severity and progression, offering valuable prognostic implications for patient management and clinical decision making. This study opens new research perspectives and suggests potential clinical implications for the treatment of patients with CF.

## Figures and Tables

**Table 1:** Baseline demographic and clinical data of the study participants *.

		CF	Healthy Controls	*p*
Sex (F/M)		42/22	20/11	
Age (years)	mean ± SD	18.02 ± 7.32	18.06 ± 5.26	0.631
	range	9–39	10–38	
BMI (kg/m^2^)	mean ± SD	19.73 ± 2.17	22.34 ± 3.13	<0.001
	range	14.4–24.6	18.3–25.6	
**Genotype**				
Homozygous ΔF508, n (%)		38 (59.3)	-	-
Heterozygous ΔF508, n (%)		26 (40.7)	-	-
2184insA, n (%)		3 (11.5)	-	-
GLy480ASp, n (%)		5 (19)	-	-
1717-1G > A, n (%)		7 (26.9)	-	-
358G > C, n (%)		5 (19)	-	-
W1282x, n (%)		4 (15.7)	-	-
N1303K, n (%)		2 (7.9)	-	-
**Clinical Laboratory Markers**				
WBC (10^3^/µL)	mean ± SD	9.97 ± 3.44	7.11 ± 2.88	0.068
	range	5.1–19.3	4.4–10.5	
NEU (%)	mean ± SD	58.2 ± 15.6	56.88 ± 9.4	0.928
	range	25.4–81.6	50.6–68.6	
CRP (mg/L)	mean ± SD	4.55 ± 3.8	2.13 ± 1.84	0.014
	range	0.5–22	0.3–4.0	
IgG (g/L)	mean ± SD	9.8 ± 2.1	9.3 ± 2.65	0.929
	range	5.7–16.3	7.8–12.3	
**Pulmonary function**				
FEV_1_ (% predicted)	mean ± SD	88.7 ± 24.3	102.16 ± 9.12	0.011
	range	35–132	98–126	

* BMI—body mass index; WBC—white blood cells; NEU—neutrophils; CRP—C-reactive protein; FEV_1_—forced expiratory volume in 1 s. Differences between means were analyzed using Mann–Whitney U tests.

**Table 2:** Spearman’s rank correlation coefficients (R) and *p*-values between CGRP concentrations and clinical features of studied patients *.

	Healthy Controls		CF	
	*R*	*p*	*R*	*p*
Age	−0.258	0.391	−0.258	0.065
BMI	−0.544	0.069	−0.147	0.299
WBC	−0.156	0.647	−0.244	0.081
NEU	−0.079	0.812	−0.261	0.061
CRP	0.354	0.301	−0.185	0.189
IgG	0.089	0.803	−0.019	0.902
FEV_1_	0.094	0.824	0.128	0.364

* BMI—body mass index; WBC—white blood cells; NEU—neutrophils; CRP—C-reactive protein; FEV_1_—forced expiratory volume in 1 s.

## Data Availability

Data available on request from the authors.
